# Are direct costs in schizophrenia influenced by duration of illness? results from a restrospective follow-up study

**DOI:** 10.1192/j.eurpsy.2024.381

**Published:** 2024-08-27

**Authors:** I. Calzavara-Pinton, G. Nibbio, B. Cabassi, E. Invernizzi, D. Di Carlo, N. Necchini, L. Bertoni, J. Lisoni, G. Deste, S. Barlati, A. Vita

**Affiliations:** ^1^DEPARTMENT OF CLINICAL AND EXPERIMENTAL SCIENCES, UNIVERSITY OF BRESCIA; ^2^Department of Mental Health and Addictions, ASST Spedali Civili di Brescia, BRESCIA, Italy

## Abstract

**Introduction:**

In Italy, it was recently estimated that the total economic burden for schizophrenia is € 2.7 billions, of which around 50% is derived from direct costs and 81% of these are due to hospitalization, residential facilities and semi-residential facilities, whereas only 10% of direct costs is derived from pharmacotherapy (Marcellusi *et al.* BMJ Open 2018; 8, e018359). Considered the high economic burden that schizophrenia has on healthcare systems (estimated to be between 1.4 % and 3 % of the total), a better characterization of the clinical variables that mostly influence the costs represent a topic of great clinical interest (Altamura *et al.* 2014 Official Journal of the Italian Society of Psychopathology 2014; 20, 223–243).

**Objectives:**

The aim of this study was to analyze whether duration of illness has an impact on the costs derived from the use of services (which account for the majority of the direct costs) in a cohort of subjects living with schizophrenia spectrum disorders (SSD).

**Methods:**

A total of 496 subjects receiving treatment from the Community Mental Health Centers (CMHC) of Brescia (Italy) were included in the study: for each patient demographic data, data regarding the duration of illness (in months), and data related to the use of service between January 1^st^, 2022 and December 31^st^, 2022 were derived from the regional database of mental health (“SIPRL”). Data on the use of service were then converted to costs using the regional rate tables for outpatient services, residential and semi-residential facilities, and the Diagnosis-Related Groups (DRG)-driven rate tables for hospitalization data. Partial correlations analyses were performed between duration of illness, corrected for age, and cost-related variables. All analyses were performed through SPSS v28 and p values <0.05 were considered significant.

**Results:**

A higher duration of illness was correlated with higher costs for outpatient non-pharmacological interventions (p=0.010), for residential facilities (p=0.025) and total costs, both including and excluding hospital admissions (p=0.005 and p=0.007, respectively), but not with hospitalization costs (p=0.773).

**Conclusions:**

The total expenditure for people living with SSD is higher for people with a longer duration of illness. These findings raise an important issue, which is that the mental health system in Italy invests more in subjects with a longer history of disease: this is in contrast with the international guidelines which prompt to intervene early in the course of the disease in patients living with SSD with outpatient rehabilitation interventions.

**Disclosure of Interest:**

None Declared

